# Posturographic analysis of induced emotion: a scoping review

**DOI:** 10.3389/fnhum.2025.1654630

**Published:** 2025-08-21

**Authors:** Adrien Hakimi, Adrien Nourry, Harold Mouras, Thierry Lelard

**Affiliations:** ^1^UR-UPJV EA 3300 APERE Adaptations Physiologiques à l'Exercice et Réadaptation à l'Effort, Université de Picardie Jules Verne, Amiens, France; ^2^Research Centre on Aging, CIUSSS de l'Estrie – CHUS, Sherbrooke, QC, Canada; ^3^Faculty of Medicine and Health Sciences, Université de Sherbrooke, Sherbrooke, QC, Canada; ^4^UR-UPJV 4559, Laboratoire de Neurosciences Fonctionnelles et Pathologies, UFR de Médecine, Université de Picardie Jules Verne, Amiens, France

**Keywords:** motor control, affective neurosciences, motivation, behavior, emotions, postural control, posturography, body sway

## Abstract

Posturography is a useful technique that allows to explore postural control, a complex motor skill enabling body orientation and stability. Analyzing postural control is one way to assess body responses to various emotional stimuli. By examining the displacement of the center of pressure, it is possible to investigate postural adjustments of individuals confronted with emotional stimuli. This scoping review focuses on the use of this methodology over the last decade. Forty-eight studies were included, covering a wide range of emotional themes studied around the world. Posturography appears to be a frequently used tool for assessing responses to emotional content. There is a high variability in the methods used in these studies and sometimes a lack of information regarding methodological concerns. This review provides methodological recommendations to standardize reporting in posturographic studies of emotions. It is likely that more and more topics will be studied in the coming years.

## 1 Introduction

Emotions are part of every individual's daily life and influence movement, just as movement can influence emotions. This relationship is necessary to allow the individuals to adapt their behavior to their environment and was already studied by (Darwin 1872). Emotions are an important modulator of an individual's motor behavior (Braine and Georges, 2023). Postural control is part of this motor behavior and is essential for performing daily tasks. This complex motor skill has two main goals: orientation for giving a frame for perception and action with respect to the external world, and stability with the regulation of the center of mass for maintaining its projection in the base of support (Massion, 1994; Horak, 2006). Like other motor skills, postural control is influenced by emotions (Adkin and Carpenter, 2018; Kosonogov et al., 2024), and postural stability has been shown to be related to emotional states and personal traits (Lebert et al., 2020). Understanding the relationship between emotional processes and bodily responses is a challenge in contemporary affective neuroscience.

The emotional response is complex, integrating both physiological parameters such as respiratory patterns, heart rate variability, and cardiorespiratory synchronization, alongside motor responses (Jerath and Beveridge, 2020). For this reason, posturography appears particularly well-suited as it seems to be influenced by the entirety of these parameters. In the absence of exogenous disturbances, postural control is influenced by mechanical parameters such as respiratory mechanics (Hodges et al., 2002), heart rate (Stürm et al., 1980), and modulation of motoneural control (Gatev et al., 1999). Posturography provides a comprehensive measure that captures the complex integration of physiological and motor responses underlying emotional states.

Force-plate and posturographic platform are the most commonly used devices to assess balance and sensori-motor control (Błaszczyk, 2016). Ground reaction forces and moments from the force plate are used to calculate the point where the plantar ground reaction force is applied, called the center of pressure (COP) (Chen et al., 2021). When maintaining a static posture, the displacement of the COP can be likened to the displacement of the human body's center of gravity (Caron et al., 2000). Analysis of COP sway, also called posturography, is considered the gold standard for assessing postural control (Chen et al., 2021) and allows to observe the result of the postural stability and the orientation necessary for interaction with the environment.

Posturography is used to assess emotional responses to various stimuli (Mouras and Lelard, 2018, 2021; Mouras et al., 2023). This non-invasive technique aims to capture subtle variations in body sway that may reflect underlying emotional processes. In a biphasic theory of emotion (Bradley et al., 2001), posturography was used to assess approach-avoidance behaviors by measuring the position of the COP when facing emotional stimuli (Mouras et al., 2023). Expected emotional responses, in this two-dimensional model, are that pleasant stimuli are associated with an approach response (appetitive behaviors), when unpleasant stimuli elicit an avoidance response (defensive behaviors). However, this model seems incomplete because on the one hand the results obtained in the face of emotional stimuli do not always go in the same direction and do not seem stereotypical (i.e., negatively valenced stimuli do not systematically lead to an avoidance response) (Carver and Harmon-Jones, 2009), and on the other hand elements seem to indicate that the reaction could be linked to the context (for example task relevance for the participant) of this interaction (Mirabella et al., 2024). Although the relationship between emotion and posture therefore appears more complex than this biphasic model and must be taken into account for the analysis of these studies, it nonetheless remains a basis for studies about emotional responses.

Among emotional behaviors, a third type of response called “freezing” has also been described. Freezing is a behavioral response corresponding to an individual's immobilization in the face of an unexpected or threatening event (Roelofs et al., 2010). This immobilization can also be described on the basis of posturographic signals and can be interpreted as a transitory, preparatory phase of the defensive response, during which the individual makes the decision to fight or flight (Azevedo et al., 2005; Roelofs et al., 2010; Hagenaars et al., 2014). By analyzing COP oscillations, freezing reactions can be identified as a reduction in the displacement of the COP.

**Figure 1:**
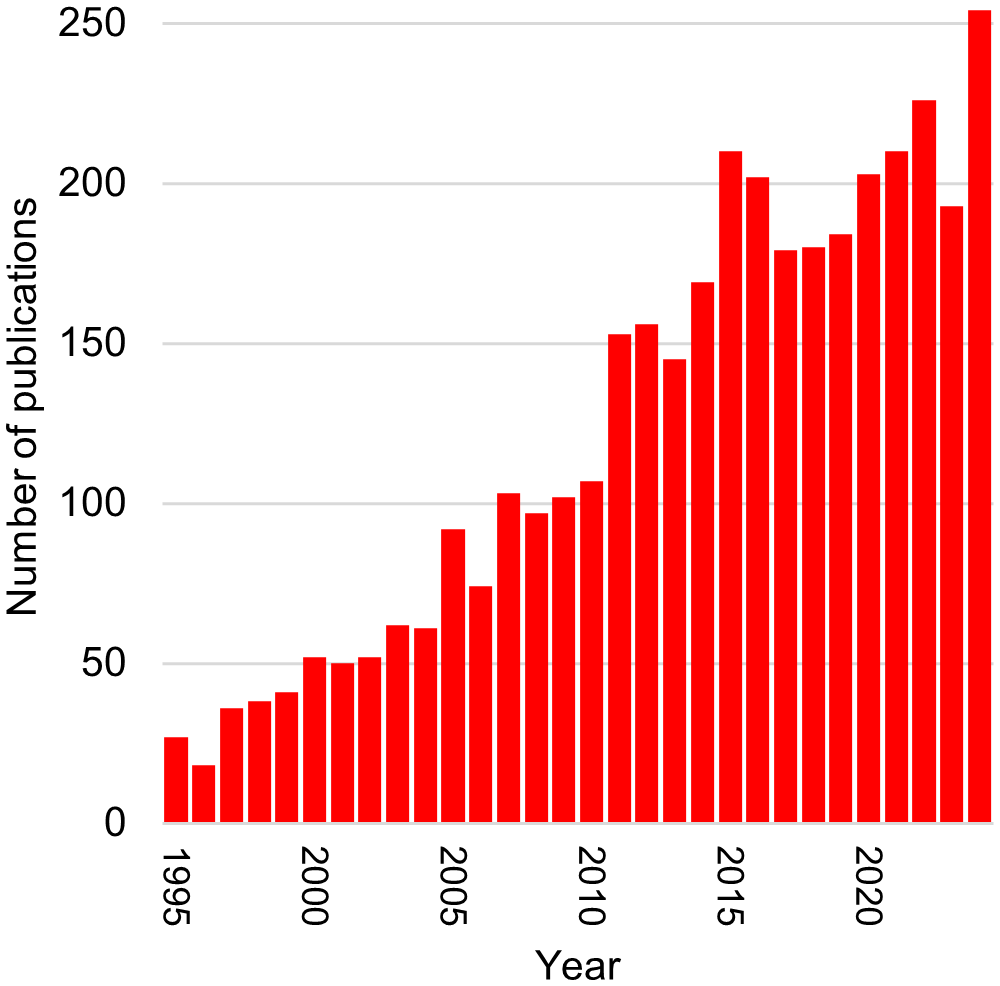
Number of studies per year for the last 30 years. This histogram shows the results of a simplified PubMed search: “postur* AND emotion,” conducted on 07/22/2025 and highlights the increasing interest in this search field.

For several decades, there has been a desire to better understand the links between emotional context and motor skills to better understand how individuals interact with their environment. During the last decade, there has been increasing interest in the posturographic assessment of emotional response of various types (Figure 1). Despite its popularity, likely due to its simplicity of use, posturography may have some limitations in interpretation, mainly because it is the result of all the movements of the body segments (Caron et al., 2000). Many parameters from these force or posturographic platforms are used to describe postural oscillations in response to emotional stimuli, so the question of the relevant parameters, as well as the most appropriate collection methodology, arises. Moreover, it is very likely that the parameters used depend on the context studied. This scoping review aims to describe the studies conducted on the use of posturography to assess emotional responses, particularly what kinds of emotional stimuli are studied and what methodological concerns are used.

## 2 Materials and methods

### 2.1 Protocol and registration

This study was carried out based on the Preferred Reporting Items for Systematic Reviews and Meta-Analyses extension for Scoping Reviews (PRISMA-ScR) guidelines (Tricco et al., 2018).

### 2.2 Eligibility criteria

The eligibility criteria have been described in the form of a PICOS table (Table 1). To be included in the review, papers had to comprise a measure of COP or center of mass (COM) in the outcomes and to use a stimulus supposed to elicit an emotional response. Only peer-reviewed journal papers were included if they were published within the last 10 years (2015–2025), written in English or French language. The review was restricted to the last 10 years to provide an overview of recent studies (supposed to be based on previous studies). Papers were excluded if they only assess dynamic task (voluntary movement of the participant, or movement induced by its environment without static measurement) or if they only compare populations without variations in the condition. Review, meta-analysis, and clinical trials were not included.

**Table 1:** PICOS description of search strategy.

**Item**	**Description**
Population	Human
Intervention	Stimulus intended to elicit an emotional response during static posturography
Comparators	At least two conditions: two different stimulus category (in terms of arousal, valence, type of stimulus) or one stimulus category with rest (including a measurement at rest).
Outcomes	Comprising COP or COM measurement
Study design	Observational studies

The terms “center of mass” and “center of mass” were included in the search strategy to allow studies that may have used a measurement strategy other than force plate to capture posturographic changes, to emerge from the search.

### 2.3 Information sources

Research was conducted on PubMed, ScienceDirect, PsycInfo, and Google Scholar and limited to the past 10 years (2015–2025). The search was last updated on 02/25/2025. References of the included articles were analyzed to search for possible other references, always limited to the range of the last 10 years.

### 2.4 Search

The research strategy used on PubMed is presented in Table 2.

**Table 2:** Research strategies.

**Databases**	**Emotion**	**Boolean operator**	**Posturography**
PubMed	((emotions[MeSH Terms]) OR (emotion^*^[Title/Abstract]) OR (sadness[Title/Abstract]) OR (fear[Title/Abstract]) OR (happiness[Title/Abstract]) OR (anger[Title/Abstract]) OR (disgust[Title/Abstract]) OR (surprise[Title/Abstract]))	AND	((center of pressure[Title/Abstract]) OR (center of mass[Title/Abstract]) OR (centre of pressure[Title/Abstract]) OR (centre of mass[Title/Abstract]) OR (posturography[Title/Abstract]))
PsycInfo	(MA emotions OR AB (emotion^*^ OR sadness OR fear OR happiness OR anger OR disgust OR surprise) OR TI (emotion^*^ OR sadness OR fear OR happiness OR anger OR disgust OR surprise))	AND	(AB (“center of pressure” OR “center of mass” OR “centre of pressure” OR “centre of mass” OR posturography) OR TI (“center of pressure” OR “center of mass” OR “centre of pressure” OR “centre of mass” OR posturography))
ScienceDirect^*^ (Title abstract keywords)	(emotion OR sadness OR fear OR happiness OR anger OR disgust OR surprise)	AND	(“center of pressure” OR posturography) (“center of mass”)
Google Scholar^**^	allintitle: emotion; emotions; emotional; fear; sadness; happiness; anger; disgust; surprise	space	“center of pressure” OR “center of mass” OR “centre of pressure” OR “centre of mass” OR posturography

* Divided in two research due to the limited number of Boolean operators.

** Divided in one research by emotion word because Google Scholar ignores parentheses.

### 2.5 Selection of sources of evidence

After removal of duplicates, eligibility for complete reading was assessed by two independent reviewers (AH, AN) based on title and abstract. Selection by the two reviewers was compared and differences were discussed, a third reviewer (TL) decided in case of disagreement. After this first selection, complete reading was processed to check eligibility and collect information.

### 2.6 Data charting process

Variables to extract were discussed among the authors. Data charting was conducted on Microsoft Excel (Microsoft Corporation, Washington, United States) by one author (AH).

### 2.7 Data items

(1) Descriptive information: authors, year of publication, title, main affiliation.

(2) Participants: number, groups, population specificity, gender, age, power analysis for sample size calculation.

(3) Emotional stimulation material: category, number, origin, description, presentation tool and/or device, subjective assessment of the material.

(4) Procedure: description, participant position.

(5) COP Measurements: measurement tool, signal processing, posturography outcomes.

(6) Other measurements: electromyography, center of mass, other.

(7) Analysis: correlation analysis, group comparison.

### 2.8 Synthesis of results

The data were analyzed, summarized, and written up by one author and then sent to the other authors for review.

## 3 Results

### 3.1 Selection of sources of evidence

**Figure 2:**
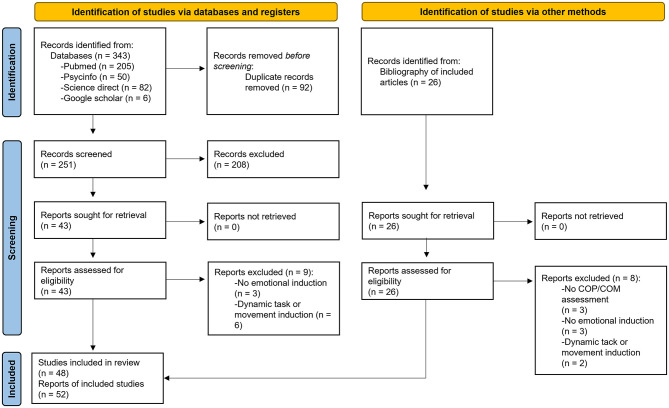
PRISMA flow diagram from Page et al. (2021). Left side shows identification via database, and right side shows identification from bibliography of included articles. The right part of each side shows the exclusions or report not retrieved. There are 52 reports about 48 studies because four reports were secondary analyses of studies' data described in previous reports. COP, center of pressure; COM, center of mass.

The study selection process is described as a PRISMA flow diagram (Page et al., 2021) in the Figure 2. After the study selection process, 52 reports about 48 studies (four reports were secondary analyses of studies' data described in previous reports) were identified. The 48 studies were included in the scoping review and are described in the Table 3. Only the first reports of each study were included, not secondary analyses. It should be noted that one study (Hiraoka et al., 2020) added new participants to a previously published dataset. As the data could not be separated, this study was fully included.

**Table 3:** Summary of included studies.

**Authors**	**Date**	**Title**	**Subject**	**Population (gender) [mean age ±SD]**	**Posturographic outcome - direction**
Akounach et al.	2022	Postural correlates of pollution perception.	Polluted environment pictures (polluted vs. non-polluted; passive observation vs. mental simulation).	31 healthy adults (14M, 17F) [25 ± 6]	Position, SD – AP
Akounach et al.	2025	Postural correlates of pleasant landscapes visual perception.	Landscape pictures (pleasant vs. neutral; passive vs. active observation)	37 healthy adults (10M, 27F) [24 ± 5]	Position, SD, length – AP/ML
Bastos et al.	2016	Stop or move: defensive strategies in human.	Gun confrontation pictures (gun vs. non-lethal object; directed on the participant vs. directed away)	71 healthy adults (36M, 35F) [23 ± 4]	SD, area – AP/ML
Beaumont et al.	2021	Postural correlates of painful stimuli exposure: impact of mental simulation processes and pain-level of the stimuli.	Painful scenes pictures (high vs. low vs. no pain; passive observation vs. mental simulation)	36 healthy adults (11M, 25F) [36 ± 11]	Position, temporal analysis – AP
Bouman and Stins	2018	Back off! The effect of emotion on backward step initiation.	Emotional pictures (erotic vs. mutilation vs. neutral) and backward step.	30 healthy adults (14M, 16F) [24 ± 3]	Length, temporal analysis – AP/ML
Brandão et al.	2016	Effects of emotional videos on postural control in children.	Emotional videos (pleasant vs. unpleasant vs. neutral), and comparison between children and adults.	23 children 7–9 years (16M, 7F) [8 ± 1] 17 children 10–12 years (8M, 9F) [11 ± 1] 19 adults (10M, 9F) [26 ± 4]	Position, length, velocity – AP/ML
Chen and Qu	2016	Influence of affective auditory stimuli on balance control during static stance.	Affective sounds (pleasant vs. unpleasant vs. neutral)	24 healthy adults (12M, 12F) [M 24 ± 2; F 23 ± 1]	Velocity, amplitude – AP/ML
Chikh et al.	2022	Perception of emotion and postural stability control at different distances.	Emotional pictures (positive vs. neutral vs. negative)	68 healthy women (68F) [21 ±1]	Position, velocity, amplitude – AP/ML
Chikh et al.	2022	How does the central nervous system control forthcoming movement with different emotional stimuli?	Emotional pictures (positive vs. neutral vs. negative) and planning a step (forward vs. backward vs. static)	18 healthy women (18F) [22 ± 2]	Velocity, amplitude – AP/ML
Ciria et al.	2017	Head movement measurement: an alternative method for posturography studies.	Emotional pictures (pleasant vs. neutral vs. unpleasant)	30 healthy women (30F) [20 ± 2]	SD, length, COP-screen distance – AP/ML
Cleworth and Carpenter	2016	Postural threat influences conscious perception of postural sway.	Postural threat (0.8 m vs. 3.2 m height) and tracking their perceived sway or not.	20 healthy adults (NaN) [21 ± 2]	RMS, frequential analysis - AP
Ellmers et al.	2022	Standing up to threats: translating the two-system model of fear to balance control in older adults	Fear of falling (ground vs. 0.6 m height) in older adults.	44 older adults (13M, 31F) [74 ± 7]	RMS, frequential analysis, sample entropy, stabilogram diffusion analysis – AP
Fawver et al.	2015	Emotional state impacts center of pressure displacement before forward gait initiation.	Emotional pictures (attack vs. sad faces vs. erotica vs. happy faces vs. neutral objects) before gait initiation.	23 healthy adults (9M, 14F) [M 20 ± 2; F 21 ± 1]	Temporal analysis – AP
Fragkaki et al.	2017	Reduced freezing in posttraumatic stress disorder patients while watching affective pictures.	Emotional pictures (pleasant vs. neutral vs. unpleasant) in posttraumatic stress disorder patients vs. healthy subjects.	14 PTSD patient (14M) [42 ± 9] 14 healthy adults (14M) [45 ± 11]	SD – AP
Gladwin et al.	2016	Ready and waiting: freezing as active action preparation under threat.	Armed threat (weapon vs. object) and potential electric shock (one opponent with shock vs. one without)	30 healthy adults (14M, 16F) [25 ± 8]	SD, temporal analysis – AP
Goulème et al.	2015	The effect of face exploration on postural control in healthy children.	Emotional faces (neutral vs. happy vs. sad vs. fear vs. angry) in three groups of children with different ages.	12 children 7–8 years (10M, 2F) [8 ± 1] 13 children 9–11 years (9M, 4F) [10 ± 1] 12 children 14–17 years (4M, 8F) [16 ± 1]	Length, area, velocity – AP/ML
Goulème et al.	2017	Postural control in children with dyslexia: effects of emotional stimuli in a dual-task environment.	Emotional faces (neutral vs. happy vs. sad vs. fear vs. angry) in children with dyslexia vs. non-dyslexic children.	22 children with dyslexia (NaN) [10 ± 0] 22 healthy children (NaN) [10 ± 0]	Length, area, velocity – AP/ML
Gui et al.	2022	Quantifying fear of falling by utilizing objective body sway measures: a 360° virtual video study.	Roller coaster videos vs. immobile room video and fear of falling.	19 healthy adults (7M, 12F) [24 ± 2]	Amplitude, RMS, frequential analysis – AP/ML
Hill et al.	2023	Effects of arm movement strategies on emotional state and balance control during height-induced postural threat in young adults.	Height (ground vs. 0.8 m) and arm position (fixed position vs. free).	30 healthy adults (18M, 12F) [22 ± 4]	RMS, frequential analysis – AP/ML
Hill et al.	2024	The influence of fear of falling on the control of upright stance across the lifespan.	Height (ground vs. 0.8 m) in three groups of different ages (children vs. young adults vs. older adults).	38 healthy children (21M, 17F) [10 ± 1] 45 young adults (26M, 19F) [22 ± 4] 15 older adults (8M, 7F) [73 ± 5]	RMS, sample entropy, frequential analysis – AP
Hiraoka et al.	2019	Differential effects of infant vocalizations on approach-avoidance postural movements in mothers.	Infant speech sounds (cry vs. laugh vs. babbling) on mothers.	20 mothers (20F) [35 ± 3]	Position, temporal analysis – AP
Hiraoka et al.	2020	Relationship between oxytocin and maternal approach behaviors to infants' vocalizations.	Infant speech sounds (cry vs. laugh vs. babbling) on mothers and oxytocin level.	39 mothers (39F) [34 ± 4]	Position – AP
Hiraoka and Nomura	2020	The influence of cognitive load on maternal postural sway and heart rate in response to infant vocalizations.	Infant speech sounds (cry vs. laugh) and cognitive task (high vs. low cognitive load) on mothers.	55 mothers (55F) [36 ± 5]	Position, temporal analysis – AP
Johnson et al.	2019	Exploring the relationship between threat-related changes in anxiety, attention focus, and postural control.	Expected movement of the platform (no threat vs. threat without perturbation experience vs. threat with perturbation experience).	80 healthy adults (30M, 50F) [22 ± 3]	RMS, frequential analysis – AP/ML
Johnson et al.	2019	Repeated exposure to the threat of perturbation induces emotional, cognitive, and postural adaptations in young and older adults.	Expected movement of the platform (threat vs. no threat) and repetition of the threat.	27 young adults (11M, 16F) [22 ± 4] 27 older adults (14M, 13F) [70 ± 4]	RMS, frequential analysis – ML
Johnson et al.	2020	The effects of distraction on threat-related changes in standing balance control.	Expected movement of the platform (threat vs. no threat) and cognitive distraction (no distraction vs. distraction).	25 healthy adults (12M, 13F) [22 ± 2]	RMS, frequential analysis – AP
Kordts-Freudinger et al.	2017	Feel bad and keep steady: emotional images and words and postural control during bipedal stance.	Emotional pictures vs. affective words, valence (positive vs. negative) and arousal (high vs. low)	45 healthy adults (22M, 23F) [24 ± 3]	Position, frequential analysis – AP
Kosonogov et al.	2024	Postural control in emotional states: an effect of biofeedback.	Emotional pictures (positive vs. neutral vs. negative) and biofeedback.	42 healthy adults (11M, 31F) [27 ± 8]	Velocity, RMS – AP/ML
Lebert et al.	2020	The impact of emotional videos and emotional static faces on postural control through a personality trait approach.	Emotional static faces vs. emotional videos with different emotions (happy vs. fear vs. angry vs. sad vs. disgust vs. neutral).	56 healthy adults (16M, 40F) [21 ± 3]	Position, SD, length – AP/ML
Lebert et al.	2021	Are you “gazing” at me? How others' gaze direction and facial expression influence gaze perception and postural control.	Emotional faces (happy vs. fear vs. angry vs. sad vs. disgust vs. neutral) and gaze direction (seven directions).	52 healthy adults (6M, 46F) [20 ± 3]	Position, area – AP/ML
Lebert et al.	2024	Keeping distance or getting closer: how others' emotions shape approach-avoidance postural behaviors and preferred interpersonal distance.	Emotional faces (happy vs. fear vs. angry vs. sad vs. disgust vs. neutral), interpersonal distance and face approach/withdrawal.	57 healthy adults (5M, 52F) [20 ± 2]	Position – AP
Lelard et al.	2017	Mental simulation of painful situations has an impact on posture and psychophysiological parameters.	Painful pictures (pain vs. no pain vs. neutral).	31 healthy adults (14M, 17F) [22 ± 4]	Temporal analysis – AP
Manetti et al.	2021	Postural effects of interoceptive imagery as a function of hypnotizability.	Verbal induction of interoception (pleasant vs. unpleasant vs. neutral).	41 healthy subjects from both genders between 20 and 30 years old	Velocity, area, length/surface ratio – AP/ML
Mouras et al.	2015	Freezing behavior as a response to sexual visual stimuli as demonstrated by posturography.	Videos (sexual vs. neutral vs. pleasant).	23 healthy men (23M) [22 ± 4]	Position, SD, area, amplitude – AP/ML
Niermann et al.	2015	Infant attachment predicts bodily freezing in adolescence: evidence from a prospective longitudinal study.	Emotional faces (happy vs. angry vs. neutral).	79 healthy adolescents (39M, 40F) [15 ± 0]	SD – AP/ML
Niermann et al.	2017	Defensive freezing links hypothalamic-pituitary-adrenal-axis activity and internalizing symptoms in humans.	Emotional faces (happy vs. angry vs. neutral) and stress induction.	92 healthy adolescents (49M, 43F) [17 ± 0]	SD – AP
Riemer et al.	2023	Emotion and motion: toward emotion recognition based on standing and walking.	Emotional videos (fear vs. sad vs. happy vs. relaxation).	24 healthy adults (10M, 14F) [25 ± NaN]	Position, SD, velocity, SD of velocity – AP/ML
Santos et al.	2019	Hands up! Atypical defensive reactions in heavy players of violent video games when exposed to gun-attack pictures.	Armed threat with pictures of a man pointing a gun vs. non-lethal object directed toward the participant.	48 healthy men (48M) [23 ± 4]	SD – AP
Stins et al.	2016	Being (un)moved by mental time travel.	Mental time travel, thinking (mental imagery) of the past and the future with different emotional valences.	32 healthy adults (16M, 16F) [20 ± 1]	Position – AP
Sturnieks et al.	2016	The influence of age, anxiety, and concern about falling on postural sway when standing at an elevated level.	Height (ground vs. 0.65 m) in older vs. young adults.	9 young adults (5M, 4F) [31 ± 5] 48 older adults (22M 26F) [76 ± 5]	Amplitude, frequential analysis – AP/ML
Takahashi et al.	2024	Effects of arousal and valence on center of pressure and ankle muscle activity during quiet standing.	Emotional pictures (pleasant vs. unpleasant vs. neutral and high vs. low arousal).	22 healthy men (22M) [24 ± 4]	SD, area, velocity, frequential analysis – AP/ML
Tashiro et al.	2025	Concern about falling is related to threat-induced changes in emotions and postural control in older adults.	Height (ground vs. 0.60 m) in older adults.	62 older adults (9M, 53F) [79 ± 6]	Position, velocity, RMS – AP
Vonesch et al.	2023	Non-linear measures of postural control in response to painful and non-painful visual stimuli.	Painful pictures (pain vs. no pain) and passive observation vs. imagery.	36 healthy adults (16M, 20F) [24 ± 6]	Sample entropy, fractal dimension, Lyapunov exponent – AP/ML
Wuehr et al.	2019	Fear of heights in virtual reality saturates 20 to 40 m above ground.	Virtual height (from 0 to 100 m) and repetition.	23 healthy adults (7M, 16F) [52 ± 16]	Velocity, RMS – AP/ML
Zaback et al.	2015	Personality traits and individual differences predict threat-induced changes in postural control.	Height (0.8 m vs. 1.6 or 3.2 m) and rest vs. rise to toes task	82 healthy adults (44M, 38F) [24 ± 4]	Position, RMS, frequential analysis – AP
Zaback et al.	2019	Adaptation of emotional state and standing balance parameters following repeated exposure to height-induced postural threat.	Height (0.8 m vs. 3.2 m) and repetition.	68 healthy adults (32M, 36F) [23 ± 4]	Position, RMS, frequential analysis – AP
Zaback et al.	2021	Selective preservation of changes to standing balance control despite psychological and autonomic habituation to a postural threat.	Height (0.8 m vs. 3.2 m) and repetition.	37 healthy adults (15M, 22F) [23 ± 4]	Position, RMS, frequential analysis – AP
Zaback et al.	2022	Facilitation and habituation of cortical and subcortical control of standing balance following repeated exposure to a height-related postural threat.	Height (0.8 m vs. 3.2 m) and repetition.	28 healthy adults (17M, 11F) [24 ± 5]	Position, RMS, frequential analysis – AP

Although studies using COM measurements could be included, most studies using it focused on dynamic tasks. Consequently, only two studies describing COM displacement were included, as they were associated with a description of COP displacement with force platforms. This combination of kinetic and kinematic parameters can provide complementary and valuable information and is described (above) in the corresponding section.

Questions were addressed by authors during the selection process and resolved after discussion. The first concerned articles introducing a sensory perturbation in the participants (tendon vibrations, cochlear stimulation). Although these stimulations were carried out without voluntary movement of the participant and can induce a fear of falling, they can cause destabilization with COP perturbation and therefore does not allow the analysis of the isolated emotional response by studying the COP. These studies were therefore not included.

Second, studies about anticipatory postural adjustments (APA), which focus on the participant's preparation for movement, have required additional eligibility criteria after discussion among the authors of the present study. APA studies that presented a COP measurement after the Go signal or that used COP data before the Go signal as a baseline only to detect the onset of movement were not included. Only four studies with a quiet standing condition or a posturographic measurement before the Go and used in the analysis for its content were included (Fawver et al., 2015; Zaback et al., 2015; Bouman and Stins, 2018; Chikh et al., 2022b).

### 3.2 Categorization by topic

**Figure 3:**
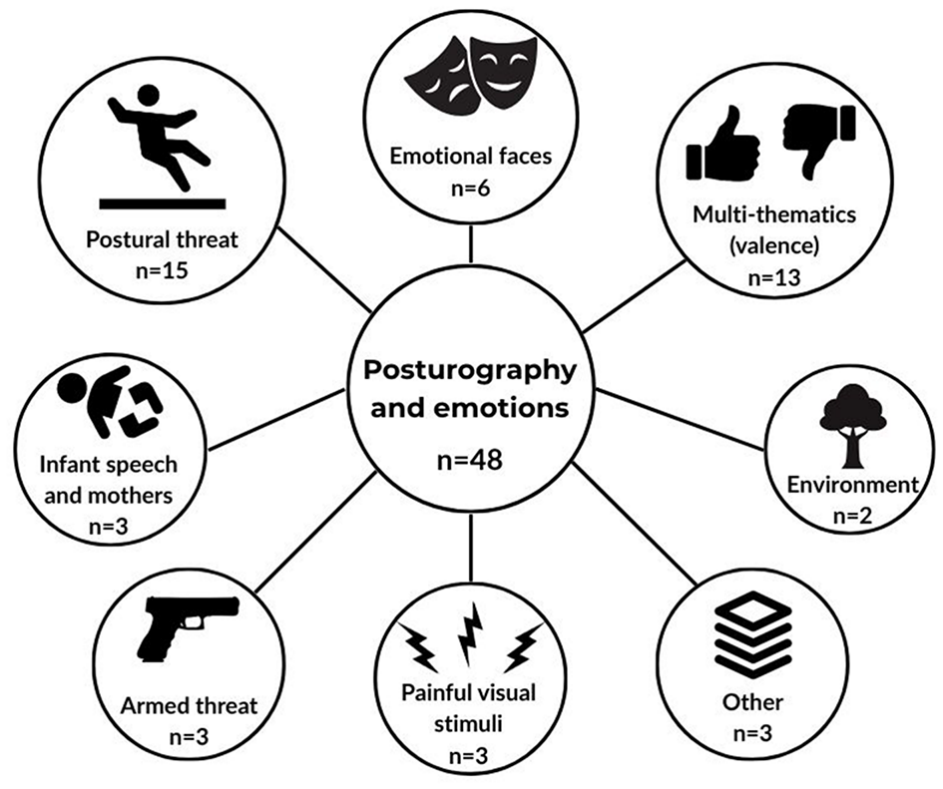
Thematic illustration.

Among the 48 articles included in this scoping review, some major topics were identified (Figure 3). Fifteen articles were about postural threat, 13 multi-thematic studies focused more on emotional valence than on a particular theme, six studies were about emotional faces, three about painful visual stimuli, three about armed threat, three about infant speech and mothers, two about environment, and three studies on unique themes were grouped as “others.”

#### 3.2.1 Postural threat (n = 15)

A common feature of postural threat studies is that they all investigate responses to the induction of fear of falling using diverse experimental methods. Among the 15 articles about postural threat, the purpose of 11 was about postural responses to height apprehension (Zaback et al., 2015; Cleworth and Carpenter, 2016; Sturnieks et al., 2016; Wuehr et al., 2019; Zaback et al., 2019, 2021a; Ellmers et al., 2022; Zaback et al., 2022; Hill et al., 2023, 2024; Tashiro et al., 2025), three about postural perturbation apprehension (Johnson et al., 2019a,b, 2020), and one about visual destabilization (Gui et al., 2022).

Numerous studies (*n* = 8) about fear of falling and height influence were conducted in Canada and share a common methodological base with the use of an hydraulic lift at 0.8 m (low condition) and 3.2 m (high condition) (Zaback et al., 2015; Cleworth and Carpenter, 2016; Zaback et al., 2019, 2021a, 2022). Interestingly, other studies about height influence used more moderate height as high condition, between 0.6 m and 0.8 m, but they all involved older adults (Sturnieks et al., 2016; Ellmers et al., 2022; Hill et al., 2024; Tashiro et al., 2025) or a more difficult standing task (Hill et al., 2023). Finally, one study used virtual reality to induce fear of height, which permits higher conditions up to 100 m (Wuehr et al., 2019).

Studies about postural perturbation apprehension were conducted with a platform on a linear positioning stage allowing controlled postural perturbations (Johnson et al., 2019a,b, 2020). Perturbations were conducted in the anteroposterior direction (Johnson et al., 2019a, 2020) or in the mediolateral direction (Johnson et al., 2019b). In these studies, during the first trial, participants were certain that the platform will not move; then, they experimented perturbations with a simulated random time before occurrence; finally, in some trials, the time before the perturbation was long enough to allow measurement while the participant waited for the perturbation.

The last study included in the postural threat topic used 360° virtual video of roller coasters (two different roller coasters and one control video in a quiet room) to induce fear of falling (Gui et al., 2022). The authors compared measurements in standing and sitting positions. Although the strategy used differs, the objective of the study remains similar to previous studies: induce fear of falling.

#### 3.2.2 Multi thematic studies (valence comparison) (n = 13)

Studies were grouped under the label “multi thematic” when they focused more on the effects of different valences (positive vs. negative vs. neutral) than on the effect of a specific theme. Valence refers to the pleasant or unpleasant nature of a stimulus and is often associated with arousal, which corresponds to the degree of excitation or physiological activation induced by a stimulus. Studies in this category are not content-centered, they mostly used in a single study a mix of themes in the following: erotic, mutilation, objects, families, babies, faces, attacks. Because they are more interested about the effects of opposite valences (and sometimes different arousals) than specific themes, two studies described in this section also used emotional faces and are therefore reported twice.

Most of the studies (*n* = 9) compared three valences (positive, neutral, negative) (Brandão et al., 2016; Chen and Qu, 2017; Ciria et al., 2017; Fragkaki et al., 2017; Bouman and Stins, 2018; Chikh et al., 2022a,b; Kosonogov et al., 2024; Takahashi et al., 2024), one study (Kordts-Freudinger et al., 2017) used two valences (positive, negative), and three studies (Fawver et al., 2015; Lebert et al., 2020; Riemer et al., 2023) used each theme separately to describe their data and therefore described more than three valences (each theme associated with a different degree of pleasantness). One study also divided the three valences into two arousal levels (Takahashi et al., 2024).

Among these studies, eight studies used pictures (Fawver et al., 2015; Ciria et al., 2017; Fragkaki et al., 2017; Bouman and Stins, 2018; Chikh et al., 2022a,b; Kosonogov et al., 2024; Takahashi et al., 2024), three videos (Brandão et al., 2016; Lebert et al., 2020; Riemer et al., 2023), one sounds (Chen and Qu, 2017), and one mixed pictures and words (Kordts-Freudinger et al., 2017). Studies using pictures (Fawver et al., 2015; Ciria et al., 2017; Fragkaki et al., 2017; Bouman and Stins, 2018; Chikh et al., 2022a,b; Kosonogov et al., 2024; Takahashi et al., 2024) mostly used IAPS (Lang et al., 1997) pictures (*n* = 7), one study (Kosonogov et al., 2024) used EmoMadrid database (Carretié et al., 2019), and the last used mixed pictures (Kordts-Freudinger et al., 2017). The number of used pictures varied from 25 to 72 with a majority of design based on three valences (positive, negative, and neutral). One study used 2 × 3 design with three valences (unpleasant vs. neutral vs. pleasant) and two arousals (high vs. low) (Takahashi et al., 2024). A specific part of this scoping review focuses on the methodology used for the presentation of images across the different topics (presented in the methodological concerns).

Videos used as emotional content were from “Film-stim” (Schaefer et al., 2010) for adults (Brandão et al., 2016; Lebert et al., 2020) and from the study of Von Leupoldt et al. (2007) for children (Brandão et al., 2016) or from movies and evaluated with a pre-study (Riemer et al., 2023). Authors used one video per emotion or valence, with a duration of 30 s (Brandão et al., 2016; Lebert et al., 2020) or longer (120–193 s) (Riemer et al., 2023).

One study (Chen and Qu, 2017) used sounds from the “IADS” database (Bradley and Lang, 2007), divided into three categories according to the subjective rating of pleasure from the database. They selected 12 pleasant, 12 neutral, and 12 unpleasant sounds of 6 s presented in condition blocks in a random order. The audio volume was chosen by the participant.

One study used affective words (*n* = 24) and pictures (*n* = 24) from various databases (Kordts-Freudinger et al., 2017). Stimuli were grouped in four categories according to their valence and their arousal. Six blocks were presented to the participant, each containing either only pictures or only words, with two stimuli from each category included in every block.

#### 3.2.3 Emotional faces (n = 6+2)

Studies about emotional faces (including two studies about mixed emotional faces and pictures already described in the multi-thematic part) investigated reactions when looking at faces displaying specific emotions (Fawver et al., 2015; Goulème et al., 2015, 2017; Niermann et al., 2015, 2017; Lebert et al., 2020, 2021, 2024). Faces were from Ekman and Friesen emotions pictures (Ekman and Friesen, 1971; Goulème et al., 2015, 2017), Ebner database (Ebner et al., 2010; Lebert et al., 2020), Karolinska Directed Emotional Faces database (Lundqvist et al., 1998; Niermann et al., 2015, 2017), and IAPS (Lang et al., 1997; Fawver et al., 2015) or virtually created for the purpose of the study and validated by a pre-study (Lebert et al., 2021, 2024). Beyond the emotional reaction to the faces, studies evaluated impact of gaze direction (Lebert et al., 2021) and interpersonal distance (Lebert et al., 2024).

#### 3.2.4 Maternal behavior facing infants' vocalizations (n = 3)

The three studies included in this topic were conducted by the same team in Kyoto, Japan (Hiraoka et al., 2019, 2020; Hiraoka and Nomura, 2020). Sounds are issued from the NTT Infant Speech Database (Amano et al., 2009) or from the Oxford Vocal Sounds database (Parsons et al., 2014). They consisted in infant laughing, crying, or babbling for 6 s (20 per condition). The protocol included interstimulus times and rest regularly. Attention was given to a maximal sound level of 70 dB and a precise description of the position of the loudspeaker. One study associated a cognitive task to the postural task (Hiraoka and Nomura, 2020), and another a hormonal measurement (Hiraoka et al., 2020). It should be noted that the study of Hiraoka et al. (2020) included the subjects of the previous study of 2019 but also involved approximately half of new participants and was therefore included in this review.

#### 3.2.5 Painful visual stimuli (n = 3)

The three studies about painful visual stimuli exposure (Lelard et al., 2017; Beaumont et al., 2021; Vonesch et al., 2023) used the same database (Jackson et al., 2005) with pictures depicting feet or hands in painful or non-painful situations. One study divided the painful condition in low pain and high pain conditions (Beaumont et al., 2021), and one study added a neutral condition with gray screens (Lelard et al., 2017). The three studies involved both passive observation and mental imagery.

#### 3.2.6 Armed threat (n = 3)

Two studies used pictures from various sources (Bastos et al., 2016; Santos et al., 2019) depicting a subject carrying either a gun or a non-lethal object. Influence of pointing direction was also tested in one study (Bastos et al., 2016). The third study (Gladwin et al., 2016) used a virtual environment with an opponent who pulls out an object (weapon or non-lethal). A second condition in this study involved the opponent, with two identifiable opponents, one of which was associated with an electric shock.

#### 3.2.7 Environment (n = 2)

The two studies about environment were conducted by the same team in Amiens, France. The first compared exposition to polluted environmental scenes and clean environmental scenes (Akounach et al., 2022), and the second compared exposition to pleasant landscape and neutral landscape (Akounach et al., 2025).

#### 3.2.8 Others (n = 3)

Two study involved mental imagery, the first about interoception (light body vs. painful body vs. neutral) (Manetti et al., 2021), the second about emotional memories or the imagination of future emotions or neutral imagery (Stins et al., 2016). Both studies involved verbal consigns.

One study specifically looked at reactions to sexual movies (Mouras et al., 2015). The authors used humorous movies to compare a non-sexual positive valence condition with neutral movies.

### 3.3 Methodological concerns

#### 3.3.1 Population

**Figure 4:**
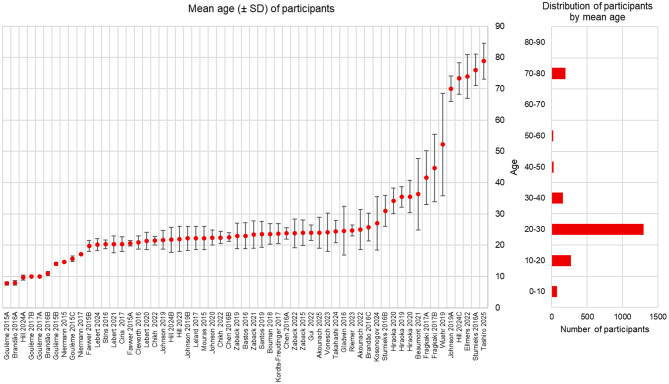
Mean age (± standard deviation) of the participants' groups in the included studies (left side) and distribution of the mean age among included studies (right side).

Across all included studies, there were 2,087 participants [813 males (39%), 1,169 females (56%), and 105 missing information). The mean study sample size was 43 ± 21. Participants involved were aged between 7.8 and 78.8 (mean age of studies), and six studies contained at least one group of children or adolescent. It should be noted that the age distribution across all studies does not cover the different decades equally (Figure 4). Most of the studies included participants between 20 and 30 (35 studies with a mean age between 20 and 30). Few studies included participants between 40 and 70 (three studies with a mean age between 40 and 70). It should be noted that only nine studies reported power analysis for sample size calculation (Kordts-Freudinger et al., 2017; Hiraoka and Nomura, 2020; Hiraoka et al., 2020; Ellmers et al., 2022; Hill et al., 2023, 2024; Lebert et al., 2024; Takahashi et al., 2024; Akounach et al., 2025) and one study conducted a sensitivity analysis (Riemer et al., 2023).

#### 3.3.2 Participant position

Feet position was imposed in 31 studies with at least one instruction on this position, and five studies reported comfortable or natural position (Stins et al., 2016; Ciria et al., 2017; Johnson et al., 2020; Gui et al., 2022; Vonesch et al., 2023). Participants were mostly barefoot (*n* = 19), some studies described participants in sockets (*n* = 1) (Ciria et al., 2017) or without shoes (*n* = 3) (Gladwin et al., 2016; Stins et al., 2016; Fragkaki et al., 2017), and one study used standardized shoes (Sturnieks et al., 2016). Feet position was reported on the platform to address reproducibility for 15 studies.

Feet were oriented at an angle of 30° in nine studies (Goulème et al., 2015, 2017; Mouras et al., 2015; Brandão et al., 2016; Lelard et al., 2017; Beaumont et al., 2021; Akounach et al., 2022; Chikh et al., 2022a,b). Nine studies (Goulème et al., 2015, 2017; Niermann et al., 2015; Brandão et al., 2016; Fragkaki et al., 2017; Chikh et al., 2022a,b; Ellmers et al., 2022; Tashiro et al., 2025) reported a standardized space between feet or heel from 2 cm to 30 cm, including five with feet oriented at 30° (space from 2 cm to 7 cm). Eleven studies (Zaback et al., 2015; Cleworth and Carpenter, 2016; Johnson et al., 2019a,b; Zaback et al., 2019; Lebert et al., 2020, 2021; Zaback et al., 2021a, 2022; Lebert et al., 2024; Takahashi et al., 2024) used anatomical references like stance width equal to their foot length or their hip width. Five studies (Bastos et al., 2016; Chen and Qu, 2017; Santos et al., 2019; Manetti et al., 2021; Hill et al., 2024) reported feet together.

Thirty studies reported arms along the body, only one study reported hands at the waist for the study purpose (Hill et al., 2024), and one study called for carrying a joystick like a weapon in front of the participant (Gladwin et al., 2016).

Eleven studies (Fawver et al., 2015; Sturnieks et al., 2016; Kordts-Freudinger et al., 2017; Niermann et al., 2017; Hiraoka et al., 2019, 2020; Wuehr et al., 2019; Hiraoka and Nomura, 2020; Riemer et al., 2023; Kosonogov et al., 2024; Akounach et al., 2025) reported no information on the participant's position or very limited information such as “stand on the board.”

#### 3.3.3 Use of pictures (methodological aspects)

Pictures were used in various topics, and by half of the studies for these reasons, the methodology of their use is the subject of this separate section. Other stimulation materials are described in the concerning topics.

Pictures were used as stimuli in 23 studies (48%) (Fawver et al., 2015; Goulème et al., 2015, 2017; Niermann et al., 2015, 2017; Bastos et al., 2016; Ciria et al., 2017; Fragkaki et al., 2017; Kordts-Freudinger et al., 2017; Lelard et al., 2017; Bouman and Stins, 2018; Santos et al., 2019; Lebert et al., 2020, 2021, 2024; Beaumont et al., 2021; Akounach et al., 2022, 2025; Chikh et al., 2022a,b; Vonesch et al., 2023; Kosonogov et al., 2024; Takahashi et al., 2024) in various themes. The databases used are described in the thematic analysis. Concerning the methodology, pictures were presented from 2 s to 25.6 s, with a majority presented for 3 s (*n* = 11) (Niermann et al., 2015, 2017; Bastos et al., 2016; Ciria et al., 2017; Fragkaki et al., 2017; Santos et al., 2019; Lebert et al., 2020, 2021, 2024; Chikh et al., 2022a,b). Most of the studies used a fixation cross (*n* = 20), sometimes before each picture (*n* = 10) (Fawver et al., 2015; Lelard et al., 2017; Bouman and Stins, 2018; Beaumont et al., 2021; Akounach et al., 2022, 2025; Chikh et al., 2022a,b; Vonesch et al., 2023; Lebert et al., 2024) or before each block (*n* = 9) (Niermann et al., 2015, 2017; Bastos et al., 2016; Ciria et al., 2017; Fragkaki et al., 2017; Santos et al., 2019; Lebert et al., 2020, 2021; Takahashi et al., 2024) or both (*n* = 1) (Kordts-Freudinger et al., 2017). Duration of the fixation cross varies from 0.5 s to 60 s, with a majority using 2 s fixation cross (*n* = 12). Most of the studies did not use interstimulus interval (*n* = 14), six studies (Goulème et al., 2015, 2017; Lelard et al., 2017; Akounach et al., 2022, 2025; Vonesch et al., 2023) used from 1- to 8-s interval between pictures, and in three studies (Fawver et al., 2015; Bouman and Stins, 2018; Chikh et al., 2022b) the interval depended of the replacement time of the participant (when a movement is required at the end of the picture). In five studies (Lelard et al., 2017; Beaumont et al., 2021; Akounach et al., 2022, 2025; Vonesch et al., 2023), participants were invited to produce mental imagery of the picture in addition to the passive observation. Pictures are mostly presented in blocks, with a majority of studies randomizing blocks order and pictures order in each block and counterbalancing it above all participants. Rest periods were used in 12 studies (Bastos et al., 2016; Lelard et al., 2017; Bouman and Stins, 2018; Lebert et al., 2020; Beaumont et al., 2021; Akounach et al., 2022, 2025; Chikh et al., 2022a,b; Vonesch et al., 2023; Kosonogov et al., 2024; Takahashi et al., 2024). Familiarization is described in seven studies (Fawver et al., 2015; Bastos et al., 2016; Fragkaki et al., 2017; Niermann et al., 2017; Bouman and Stins, 2018; Chikh et al., 2022a,b) using pictures.

Various software were used to present the pictures, but only 10 studies (43%) reported the software used (Fawver et al., 2015; Bastos et al., 2016; Ciria et al., 2017; Lelard et al., 2017; Lebert et al., 2021, 2024; Akounach et al., 2022, 2025; Chikh et al., 2022a; Vonesch et al., 2023). These software were E-Prime (*n* = 5, Psychology Software Tool); Open Sesame [*n* = 2, Open source (Mathôt et al., 2012)]; LabVIEW (*n* = 1, National Instrument); Pinnacle (*n* = 1, Corel); and Presentation (*n* = 1, Neurobehavioral system). Most of the studies used a screen (*n* = 19), but a small part also used video projector (*n* = 4). The size of the screen or projection was reported in 14 studies. Dimensions varied widely, ranging from 17 inches to 2 m × 1.5 m. Three studies reported the visual angle (Bastos et al., 2016; Fragkaki et al., 2017; Lebert et al., 2020), and two reported the image size (Fawver et al., 2015; Bouman and Stins, 2018). Seven studies described the resolution of the screen or projection used (Fawver et al., 2015; Goulème et al., 2015, 2017; Kordts-Freudinger et al., 2017; Lebert et al., 2020, 2021, 2024).

The distance between the participant and the screen was reported in 19 studies. One study (Chikh et al., 2022a) used this distance as a variable, and three studies (Fawver et al., 2015; Lelard et al., 2017; Santos et al., 2019) provided no information about this. The distance ranged from 0.3 to 6 m, with a majority of studies using a distance of 1 m between participant and screen (*n* = 9). It should be noted that of the four studies using a video projector (Fawver et al., 2015; Lelard et al., 2017; Chikh et al., 2022a,b), only one gave the distance as 6 m (Chikh et al., 2022b), and a second used the distance as a variable.

#### 3.3.4 Subjective assessment of the material

Subjective assessment of the stimulation material allows investigators to verify that the material was perceived as expected by the participant. A subjective assessment of the stimulation material was described in 39 studies (81%). On these, 33 assessed each stimulus independently when four assessed blocks (Bastos et al., 2016; Lebert et al., 2020, 2021, 2024), one study (Gladwin et al., 2016) assessed the condition factors, and a last study (Niermann et al., 2017) conducted assessment of stress in a more timed manner.

Concerning the 33 assessing each stimulus, the timing was for 18 (Zaback et al., 2015; Brandão et al., 2016; Cleworth and Carpenter, 2016; Stins et al., 2016; Sturnieks et al., 2016; Johnson et al., 2019a,b; Wuehr et al., 2019; Zaback et al., 2019; Hiraoka and Nomura, 2020; Johnson et al., 2020; Zaback et al., 2021a; Ellmers et al., 2022; Gui et al., 2022; Zaback et al., 2022; Hill et al., 2023, 2024; Tashiro et al., 2025) after each stimulus, 13 (Niermann et al., 2015; Ciria et al., 2017; Fragkaki et al., 2017; Bouman and Stins, 2018; Hiraoka et al., 2019, 2020; Beaumont et al., 2021; Manetti et al., 2021; Akounach et al., 2022, 2025; Riemer et al., 2023; Kosonogov et al., 2024; Takahashi et al., 2024) after the measurement session, and two (Kordts-Freudinger et al., 2017; Chikh et al., 2022a) after each block.

A thematic trend can be observed: studies on postural threat (height, destabilization) all favor assessment immediately after each condition. Studies using exposure to photographs preferentially use an assessment after the measurement's session (*n* = 9) or an assessment after each block of photographs (*n* = 2).

#### 3.3.5 Task relevance

The relevance of the task assigned to participants can influence the results obtained (Mirabella et al., 2024). Thirty-four studies used passive exposition to the stimulus (i.e., passive observation, passive earing, and passive exposition to height) and therefore did not include a task to be carried out by the participant. Of the 14 studies that included a task, eight can be considered relevant to the emotional stimulus, including seven involving mental imagery (Stins et al., 2016; Lelard et al., 2017; Beaumont et al., 2021; Manetti et al., 2021; Akounach et al., 2022, 2025; Vonesch et al., 2023) and one shooting task (Gladwin et al., 2016). Regarding the other studies whose task was not related to the emotional stimulus, three studies involved preparation for a movement at picture offset (Fawver et al., 2015; Bouman and Stins, 2018; Chikh et al., 2022b), two used a sequence of numbers or letters memorizing (Hiraoka and Nomura, 2020; Johnson et al., 2020), and one used anteroposterior postural tracking (not directly related to the height condition) (Cleworth and Carpenter, 2016).

#### 3.3.6 Analysis

Correlation analysis involving COP measurements was conducted in nine studies (Niermann et al., 2015; Ciria et al., 2017; Hiraoka et al., 2019; Johnson et al., 2019a; Wuehr et al., 2019; Hiraoka and Nomura, 2020; Akounach et al., 2022, 2025; Lebert et al., 2024). Associations between COP measurements and subjective rating were tested in four studies, between COP measurements and questionnaires in four studies, between COP measurements and other physiological measurements in six studies, and between COP measurement and preferred distance with faces in one study. Group comparison was conducted in 12 studies, with five about age (Goulème et al., 2015; Brandão et al., 2016; Sturnieks et al., 2016; Johnson et al., 2019b; Hill et al., 2024), two comparing pathological condition with control group (Fragkaki et al., 2017; Goulème et al., 2017), two according fear of falling (Ellmers et al., 2022; Tashiro et al., 2025), one according to the infant–parent relationship (Niermann et al., 2015), one according to hypnotizability (Manetti et al., 2021), and one according to the video game playing frequency (Santos et al., 2019).

### 3.4 Posturographic assessment

Various force plates were used in studies from home made to well-known devices. Interestingly, there is a wide range of sample frequencies from 40 Hz to 5,000 Hz. Some authors (*n* = 3) down-sampled their data (to 25, 100, or 200 Hz) (Ciria et al., 2017; Niermann et al., 2017; Akounach et al., 2025). Most studies (*n* = 23) reported the use of a low-pass filter on their posturographic data with mostly 5 Hz (*n* = 10) or 10 Hz (*n* = 10) cutoff frequencies (Zaback et al., 2015; Bastos et al., 2016; Brandão et al., 2016; Cleworth and Carpenter, 2016; Chen and Qu, 2017; Fragkaki et al., 2017; Kordts-Freudinger et al., 2017; Niermann et al., 2017; Johnson et al., 2019a,b; Santos et al., 2019; Wuehr et al., 2019; Zaback et al., 2019; Johnson et al., 2020; Zaback et al., 2021a; Ellmers et al., 2022; Zaback et al., 2022; Hill et al., 2023; Riemer et al., 2023; Hill et al., 2024; Takahashi et al., 2024; Akounach et al., 2025; Tashiro et al., 2025). One of them also used a high-pass filter with a cutoff frequency at 0.1 Hz (Niermann et al., 2017). It is important to note that 18 studies do not provide any information on possible data processing, and one study reported the use of a low-pass filter without frequency specification (Kordts-Freudinger et al., 2017). Three studies described elimination of outliers >3SD (Fawver et al., 2015; Hiraoka and Nomura, 2020; Hiraoka et al., 2020), four studies (Stins et al., 2016; Bouman and Stins, 2018; Hiraoka et al., 2019; Hiraoka and Nomura, 2020) used means (0.5 s, 1 s, or 5 points), and one study (Gladwin et al., 2016) used sliding window (2 s shifted by 0.1 s).

Part of the studies (*n* = 18) reported recalibration of the data using the mean COP position of the full record (*n* = 9) or a various time before stimulus presentation (*n* = 7, from 500 ms to 6 s) (Fawver et al., 2015; Cleworth and Carpenter, 2016; Gladwin et al., 2016; Stins et al., 2016; Ciria et al., 2017; Kordts-Freudinger et al., 2017; Hiraoka et al., 2019; Johnson et al., 2019a,b; Zaback et al., 2019; Hiraoka and Nomura, 2020; Johnson et al., 2020; Zaback et al., 2021a; Akounach et al., 2022; Chikh et al., 2022a; Zaback et al., 2022; Hill et al., 2023; Tashiro et al., 2025). One study (Hiraoka and Nomura, 2020) used the first value of the record, and one study did not precise the technique used (Stins et al., 2016).

**Figure 5:**
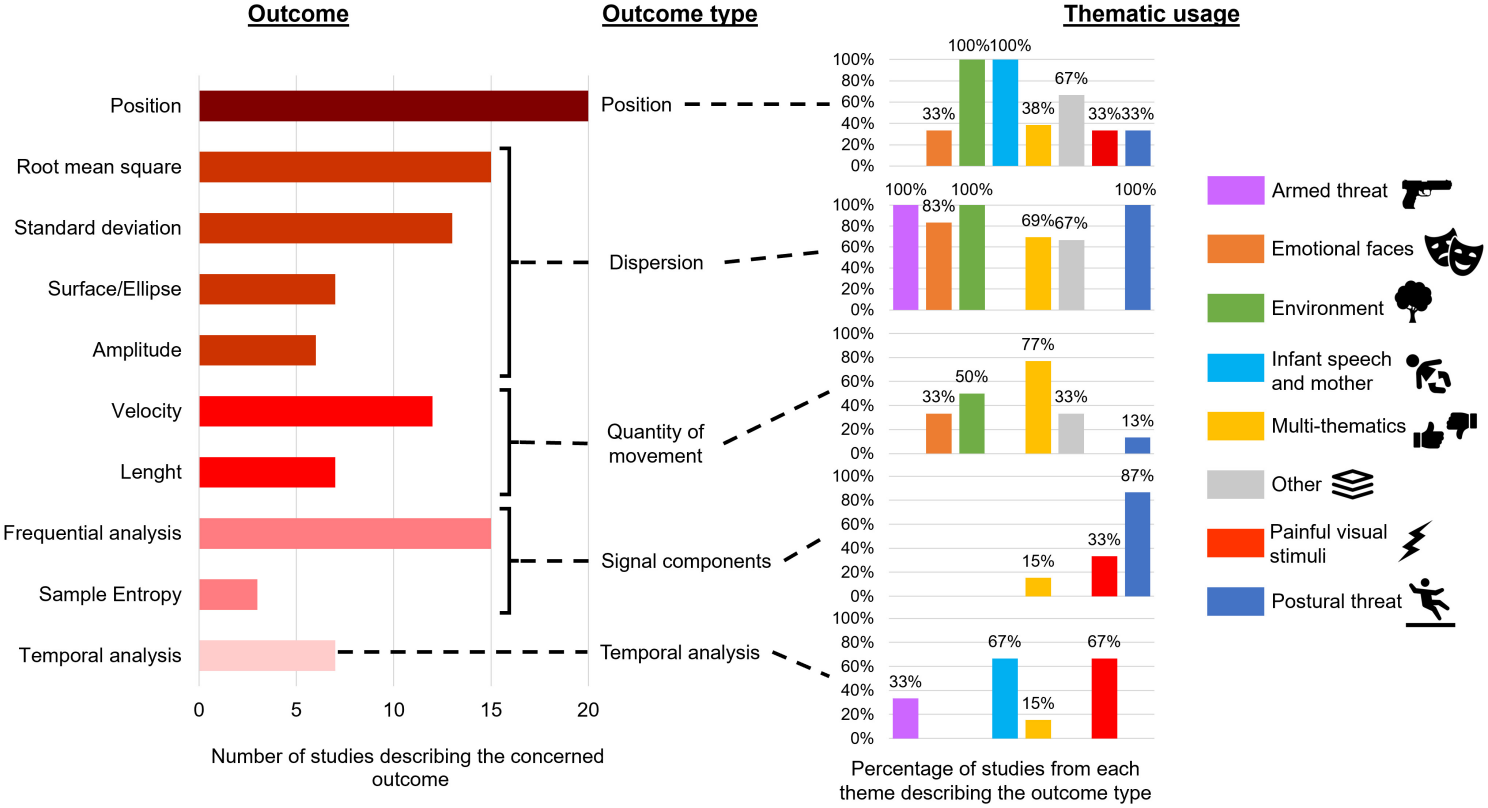
Number of studies using the outcomes and distribution among themes.

Concerning the outcomes, half of the studies used only one dimension, with a large majority about the antero-posterior dimension (*n* = 23, 50%) and only one study (Johnson et al., 2019b) about the mediolateral dimension (2%). The second half of the studies used both dimensions, at least partially, in the outcomes (*n* = 24, 50%). The type and proportion of the most frequents outcomes used is described in Figure 5. Other outcomes described are distance between the screen and the COP, stabilogram diffusion analysis, length/surface ratio, velocity standard deviation, fractal dimension, and Lyapunov exponent.

Two studies also used a motion capture system to assess the COM. The first used an Optotrak system (Northern Digital Inc., 100 Hz) with five markers to assess the COM (Cleworth and Carpenter, 2016), while the second used 14 cameras Oqus 300 and Oqus 500 (Qualisis, 120 Hz) with 32 markers (Riemer et al., 2023). Finally, a third study simultaneously studied participants' head movements using a standard webcam (25 Hz) (Ciria et al., 2017).

### 3.5 Physiological assessment

Physiological measurements were carried out by 23 studies. A large part recorded electrodermal activity (*n* = 13) (Zaback et al., 2015; Cleworth and Carpenter, 2016; Sturnieks et al., 2016; Lelard et al., 2017; Johnson et al., 2019a,b; Wuehr et al., 2019; Zaback et al., 2019; Johnson et al., 2020; Zaback et al., 2021a; Akounach et al., 2022; Zaback et al., 2022; Akounach et al., 2025) and/or heart rate (*n* = 12) (Niermann et al., 2015, 2017; Bastos et al., 2016; Gladwin et al., 2016; Sturnieks et al., 2016; Fragkaki et al., 2017; Lelard et al., 2017; Wuehr et al., 2019; Hiraoka and Nomura, 2020; Manetti et al., 2021; Akounach et al., 2022; Takahashi et al., 2024). Six studies recorded electromyography (Sturnieks et al., 2016; Wuehr et al., 2019; Zaback et al., 2019, 2021a, 2022; Takahashi et al., 2024), all of them for the tibialis anterior, five of them for the soleus (Wuehr et al., 2019; Zaback et al., 2019, 2021a, 2022; Takahashi et al., 2024), and one study for the medial gastrocnemius (Sturnieks et al., 2016). Four studies calculated the co-contraction index from antagonist muscular activity (Wuehr et al., 2019; Zaback et al., 2019, 2021a, 2022). Two studies used eye-tracking (Goulème et al., 2015, 2017). Two other involved hormonal saliva measurement (Niermann et al., 2017; Hiraoka et al., 2020). Two studies assessed blood pressure (Sturnieks et al., 2016; Niermann et al., 2017). One study used a pneumogram (Manetti et al., 2021). One study assessed limits of stability (Tashiro et al., 2025). Finally, one study used an electro-encephalogram (Zaback et al., 2022).

## 4 Discussion

The objective of this scoping review was to establish an inventory of research carried out with posturography on emotional reactions over the last 10 years. This study highlights the growing interest in the use of posturography in the study of emotional reactions. A wide variety of topics have been studied, and many more will likely be explored in the future.

### 4.1 Highlights

This review, based on methodology rather than on a particular theme, has made it possible to draw up a broader inventory of the methods used to acquire and analyze emotional postural responses in the different themes. Eight themes were identified in this study: postural threat, multi-thematic (valence based), emotional faces, maternal behavior facing infants' vocalizations, painful visual stimuli, armed threat, environment, and “others.” During the screening of literature, other themes were found such as age-stereotype threat. These references were not included in this scoping review because they only involved dynamic tasks and not static posturography. The objective was to review studies that use posturography to study individuals' emotional responses, not the influence of emotions on movement. As numerous references describing the influence of emotions on motor behavior during dynamic tasks were excluded, it could be the subject of another scoping review.

### 4.2 Methodological concerns

**Figure 6:**
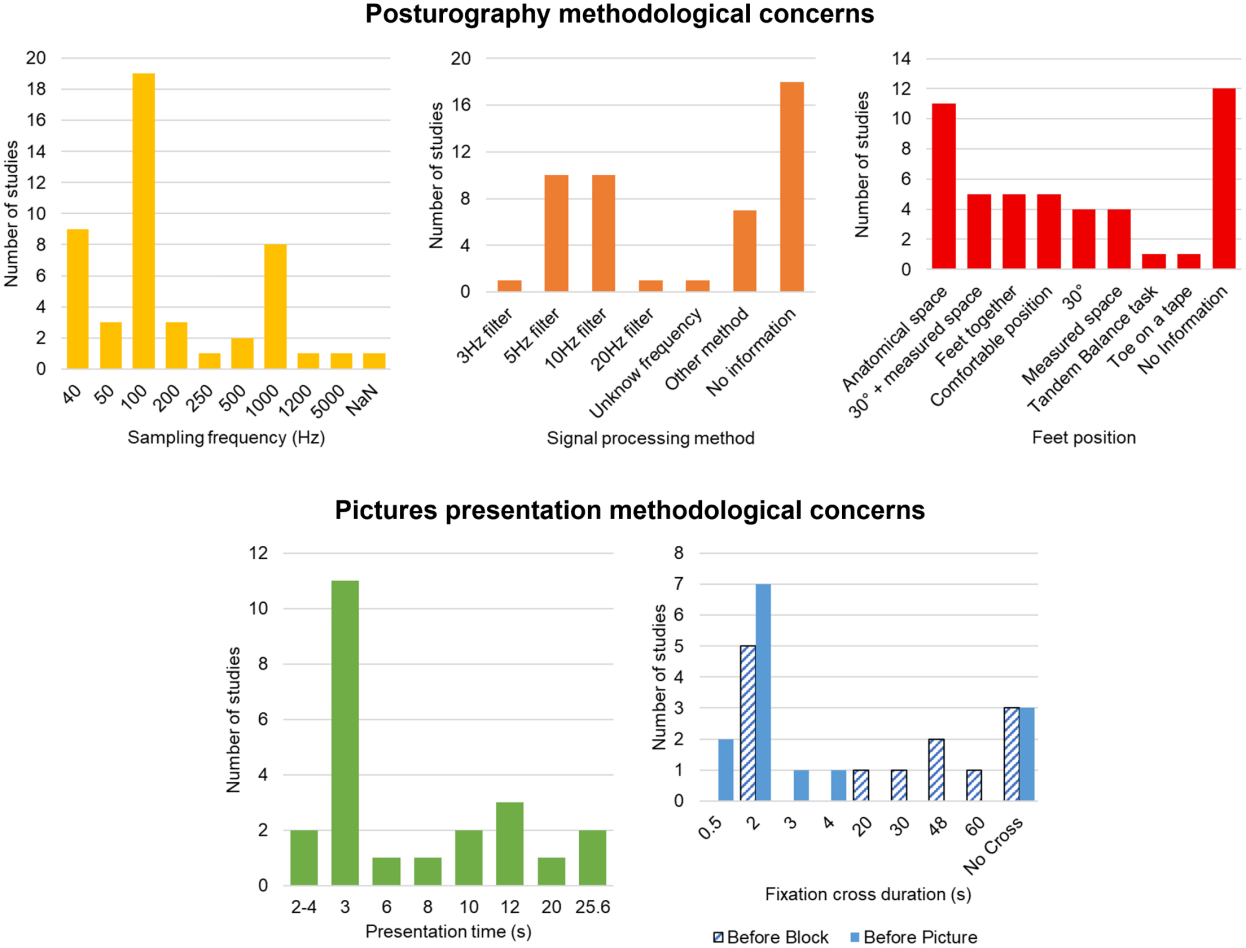
Illustration of the disparity between studies concerning the methodology.

The main highlight of this scoping review is the wide variability of methodological concerns (Figure 6). The position of participants is a source of inconsistency among studies as it was previously reported (Julienne et al., 2024). It should be noted that information about participant position is not always reported in full which is a significant gap for reproducibility. For example, it is well established that variations in foot position can lead to significant differences in results, and whatever method is used, it should be reported (Gibbons et al., 2019). Position is not the only source of differences among studies. Sampling frequency is probably the most representative inconsistent methodological element, with nine different frequencies among studies from 40 Hz to 5,000 Hz. A very large variability was also observed in the filtering applied to the data, particularly concerning the cutoff frequency used for the filter. Furthermore, the lack of information provided on signal processing by 18 studies is a major element to improve for future studies in the age of open science, especially since it is clearly established that these elements can significantly influence the results (Schmid et al., 2002). The low rate of description of calculation methods for obtaining COP parameters and the extensive heterogeneity in methodological characteristics were already highlighted by authors (Julienne et al., 2024).

As highlighted in Figure 4, there is a lack of studies about people between 40 and 70 years old. This is probably due to a convenience sampling with easier recruitment of university student (Nielsen et al., 2017), but this should be taken into consideration for future studies, especially because age is an important factor influencing postural control (Julienne et al., 2024).

Approximately 20% of studies did not use a subjective rating of the stimulus. Stimulus subjective evaluation is a key component of the protocol that allows us to determine whether the stimulus was perceived as expected by the participants. Analyses of how emotions impact behavior require controlling not only the valence of the stimuli but also their arousal levels. The lack of stimulus evaluation makes it difficult to interpret the results. One of the most used methods to measure participants' perception of the stimulus is the self-assessment manikin (Bradley and Lang, 1994), a pictorial questionnaire used to rate valence, arousal, and dominance. If not applicable, the use of a scale (visual or numerical) to define whether the stimulus was perceived by the participant as expected by the investigator is necessary.

Beyond the subjective evaluation of the material, recent evidence seems to support the importance of context and, in particular, the relevance of the proposed task (Mirabella et al., 2024). It therefore seems important for future studies to take into account the relevance of the task for the design of protocols and the interpretation of results.

The majority of studies have focused on anteroposterior oscillations, consistent with the search for approach-avoidance behaviors (Mouras et al., 2023). Far fewer studies have focused on mediolateral oscillations. In a behavioral context, however, mediolateral oscillations could provide important information about a decrease in oscillations indicative of freezing behavior (Robillard et al., 2011). Frequency analyses have been conducted primarily in studies of postural threat. A few other indices, often associated with more complex mathematical analyses, have been used more occasionally.

### 4.3 Use of pictures

Nearly half of the studies used picture presentation to induce an emotional response; however, regarding the use of pictures in studies, there is a great disparity in the methodology used for the presentation of images (presentation time, interstimulus time, fixation cross, familiarization, etc.). A large variability in the distance between the participant and the screen was also found in the studies. The use of the visual angle proposed by some authors (Bastos et al., 2016; Fragkaki et al., 2017; Lebert et al., 2020) seems an interesting compromise, allowing a simple comparison of the different methods. Although studies using pictures presentation may focus on different themes, the type of stimulation material used (i.e., pictures) remains similar, and it would be interesting to establish optimal criteria for picture presentation to obtain the best emotional response. For example, what is the optimal time to capture the participant's emotional response, and from what time do we observe a secondary response more linked to a cognitive analysis of the image? Similarly, from what duration of presentation of successive images do we observe a component of fatigue and should we therefore offer a break? These parameters should also take into account the use of passive observation or mental imagery.

### 4.4 Physiological assessment

The secondary physiological measures conducted in these studies were mainly electrodermal activity and heart rate. These have been widely used in studies of emotions and are primarily reflections of autonomic nervous system activity (Horvers et al., 2021; Gullett et al., 2023). They are used to validate the occurrence of an emotional change during the stimulus. Another fairly widespread measure is the use of electromyography on the antagonist muscles of the leg. The exploration of the activity of these muscles aims to look for stiffening of the ankle through co-contraction of the antagonists. The use of other measures in parallel with posturography makes it possible to reinforce the results obtained when the different measures converge toward the same response.

### 4.5 Recommendations

Some recommendations may arise from this study regarding the information to be reported in studies about posturographic analysis of emotions. The participant's position must be clearly defined, including whether it is free. Otherwise, authors should report at least the position of the feet and any other specific instructions. Subjective evaluation of the stimulation material should be systematically conducted and reported, either with the self-assessment manikin, or, if not applicable, with standardized tools to ensure stimuli are perceived as intended, thereby controlling for both valence and arousal levels to enable meaningful interpretation of results. Protocols should be designed and the results are interpreted taking into account the relevance of the task. Familiarization should be encouraged and reported to limit the first-trial effect (Zaback et al., 2021b). Sampling frequency and signal processing (at least recalibration and filtering) must be systematically reported. Mediolateral COP data analysis should be encouraged, or if it yields no results, it should be specified in the report that these data were analyzed without interpretable results. Regarding the use of images, the following parameters should be given in the method if applicable: presentation time, use and duration of a fixation cross, interstimulus time, possible break, presentation software, and visual angle (or elements allowing it to be calculated, i.e., distance between the participant and the screen and dimensions of the image presented). It is also important to remember that posturography provides information on the result of postural control, therefore both postural stability and orientation, which must be taken into account when analyzing its results.

The data extracted in this scoping review can guide researchers who would like to use posturography to assess the behavioral response to an emotional stimulus, on the methodology to use.

### 4.6 Limitations

This review has some limitations: it was limited to the last 10 years and to four databases; only one author carried out the data collection, although inclusion was assessed by several authors; it did not include dynamic task, and it did not address the results of the studies.

## 5 Conclusion

The use of posturography in the study of behavioral response to emotional stimuli has grown over the last decade and seems to be increasingly used. The present study pointed out a great variability in the methodology used. Future studies should focus on describing all methodological elements to allow for greater reproducibility.
